# Reconstructing Superquadrics from Intensity and Color Images

**DOI:** 10.3390/s22145332

**Published:** 2022-07-16

**Authors:** Darian Tomašević, Peter Peer, Franc Solina, Aleš Jaklič, Vitomir Štruc

**Affiliations:** 1 Faculty of Computer and Information Science, University of Ljubljana, 1000 Ljubljana, Slovenia; peter.peer@fri.uni-lj.si (P.P.); franc.solina@fri.uni-lj.si (F.S.); ales.jaklic@fri.uni-lj.si (A.J.); 2 Faculty of Electrical Engineering, University of Ljubljana, 1000 Ljubljana, Slovenia; vitomir.struc@fe.uni-lj.si

**Keywords:** superquadrics, reconstruction, color images, deep learning, convolutional neural networks

## Abstract

The task of reconstructing 3D scenes based on visual data represents a longstanding problem in computer vision. Common reconstruction approaches rely on the use of multiple volumetric primitives to describe complex objects. Superquadrics (a class of volumetric primitives) have shown great promise due to their ability to describe various shapes with only a few parameters. Recent research has shown that deep learning methods can be used to accurately reconstruct random superquadrics from both 3D point cloud data and simple depth images. In this paper, we extended these reconstruction methods to intensity and color images. Specifically, we used a dedicated convolutional neural network (CNN) model to reconstruct a single superquadric from the given input image. We analyzed the results in a qualitative and quantitative manner, by visualizing reconstructed superquadrics as well as observing error and accuracy distributions of predictions. We showed that a CNN model designed around a simple ResNet backbone can be used to accurately reconstruct superquadrics from images containing one object, but only if one of the spatial parameters is fixed or if it can be determined from other image characteristics, e.g., shadows. Furthermore, we experimented with images of increasing complexity, for example, by adding textures, and observed that the results degraded only slightly. In addition, we show that our model outperforms the current state-of-the-art method on the studied task. Our final result is a highly accurate superquadric reconstruction model, which can also reconstruct superquadrics from real images of simple objects, without additional training.

## 1. Introduction

Scene reconstruction from visual data represents a fundamental field of research in computer vision. Its main goal is to reconstruct an observed environment, as accurately as possible, by describing the various objects in the scene. One of the prevalent reconstruction approaches relies on representing complex scenes via a set of simple geometric shapes, also known as volumetric primitives (the most expressive and versatile of these primitives are currently superquadrics) [1,2]. Since the number of reconstructed primitives can be adjusted, this allows for a rather flexible and detailed solution to the problem. Successfully reconstructed environments can then be used by autonomous agents for various tasks, such as navigating their surroundings [3,4] or grasping objects [5,6], both of which have practical applicability, e.g., in warehousing and manufacturing.

The outlined reconstruction approach, which relies on volumetric primitives, is commonly known as bottom-up reconstruction. It was first introduced to vision systems by Marr [7], whose theoretical vision system utilized various types of depth information to fit appropriate volumetric models in a hierarchical manner. The transition from theoretical systems to practical applications occurred much later and was strongly influenced by the choice of 3D representations. Since detailed representations required a large number of parameters and, thus, more complex models, it was clear that representations with fewer parameters were necessary. Representations, such as shape primitives, would thus allow for less complexity at the expense of reconstruction accuracy, which can often be minimal. Following this train of thought, superquadrics (a class of volumetric primitives) were introduced to computer graphics by Barr [1]. The idea of superquadrics was then carried over to computer vision by Pentland [8]. More formally, superquadrics are 3D models that require only a few shape parameters to form a variety of different shapes, with other parameters describing their size as well as position and rotation in space.

After a long hiatus, the topic of superquadric recovery was revisited, inspired by tremendous advances in deep learning. More recent works relied on the use of convolutional neural networks (CNNs) to recover shape primitives from a scene [9,10,11,12,13,14,15]. These state-of-the-art approaches bypass the computational overhead of early iterative solutions and exhibit considerably higher reconstruction accuracy. They also address reconstruction from different types of data, such as point clouds, depth images, and even a combination of RGB images and mesh data. To achieve successful reconstructions, all approaches adopt learning objectives, which include a certain level of geometric information. Recently, Oblak et al. [13] moved past the constraints of 3D data and showcased that reconstruction of superquadrics from a single depth image is possible with the use of deep learning. The authors relied on a CNN predictor, trained with a custom geometry-based loss, to estimate the size, shape, position, and rotation of a depicted superquadric.

While modern superquadric recovery approaches do achieve incredible reconstruction accuracy, they remain limited in terms of input data (to point cloud data or depth images). Unfortunately, despite advancements in sensor technologies, such types of data remain quite difficult to obtain, especially for arbitrary tasks or situations. This, in turn, significantly limits the applicability of these reconstruction methods. Meanwhile, mechanisms for gathering RGB images are already prevalent and could be easily exploited given a suitable reconstruction approach.

In this study, we address the need for an RGB-based superquadric recovery solution, which is capable of reconstructing unknown objects in an uncontrolled environment. To achieve this, we followed the general idea of recent methods [12,13] based on depth images but took a step further and explored the usage of deep learning models for reconstruction of superquadrics from a single RGB image. The main challenge we faced: RGB images lack the invaluable spatial information provided by depth images, which is extremely important for correctly predicting the position of superquadrics in space. We took a gradual approach to solve the reconstruction task, by training and evaluating the predictor on increasingly complex images. We propose two methods for dealing with the lack of spatial information in RGB images. The first method is based on fixing the *z* position parameter of the superquadrics, which in turn removes the ambiguity in (superquadric) position and size. The second method relies on the addition of shadow-based cues to superquadric images to obtain the required spatial information. For this approach, we drew inspiration from similar works that leveraged shadow-based cues to recover the shapes or sizes of objects [16,17].

To facilitate this study, we experimented with fixed-sized images (i.e., 256×256 pixels), which included only one superquadric, allowing us to focus only on the reconstruction task. We first limited ourselves to simple intensity images with gray superquadrics on a black background and then moved to RGB images with randomly colored or textured superquadrics and backgrounds. To evaluate our results, we used both qualitative and quantitative techniques in the form of visualized reconstructions of superquadrics as well as error distributions of superquadric parameters and accuracy distributions of the predictions. We also compared our results with results based on Oblak et al. [13] and reflected on the differences between the analyzed problems. In addition, we compared our method to the state-of-the-art solution for a similar task, proposed by Paschalidou et al. [10].

**Figure 1:**
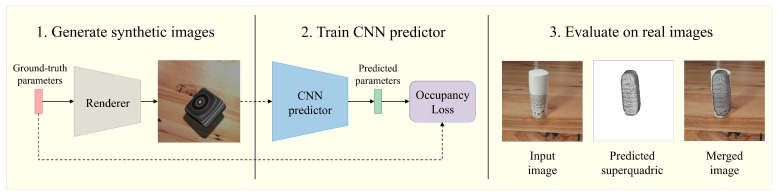
Visualized overview of our reconstruction method. (1) Generate a large dataset of synthetic images that mimic the real environment. Each image contains a randomly positioned superquadric of a random shape. (2) Train the CNN predictor on the synthetic dataset to predict superquadric parameters. (3) Use the final CNN predictor (*Textured on Wood (Fixed z) with Sh. & S.L.*) on real images to reconstruct simple objects as superquadrics.

Previous superquadric recovery approaches have already shown promising results in practical applications, most notably in robot grasping tasks [18,19,20,21]. Other interesting applications include handling of mail pieces [22], documentation of artifacts [23,24], and recently, the representation of the human body [25]. However, these practical applications are again based on point cloud data or depth images. By successfully recovering superquadrics from a single RGB image, we aim to widen the use and applicability of superquadric reconstruction approaches, due to the mass availability of such data. In our work, we also avoid the need for manually labeled real-world data, which is difficult to obtain, by training and testing the CNN predictor on synthetic images and later observing how well the model generalizes to real images. An overview of our method and the discussed phases are present in Figure 1. As we show, we are able to approximate various simple real-world objects with superquadrics, from just a single real-world RGB image, without any camera calibration. We believe that this approach could be tremendously useful for future robot-grasping approaches, especially when the object shapes are not known in advance, since it requires minimal human interaction.

The main contributions of this paper are as follows:

- We extend previous superquadric reconstruction approaches from depth images to intensity and color images, and show that even in this challenging setting, comparable reconstruction quality can be achieved.
- We propose two data modification methods to combat the lack of spatial information, which helps to reduce the parameter ambiguity and allows for a successful estimation of the superquadric parameters. The first includes fixing the *z* position parameter of the generated superquadrics, whereas the second relies on the addition of shadow cues to the images.
- We demonstrate that our CNN predictor outperforms current state-of-the-art methods and is also capable of generalizing from synthetic to real images.

## 2. Related Work

In this section, we briefly discuss existing superquadric reconstruction techniques and then provide an overview of closely related deep learning methods based on 3D data.

### 2.1. Superquadric Recovery

The process of superquadric recovery entails estimating superquadric parameters (size, shape, position, and rotation) from the given input data so that the reconstructed superquadric fits the data as closely as possible.

**Early Methods.** In the past, a significant amount of research was dedicated to studying the reconstruction of superquadrics from 3D data. For recovering superquadrics from color images, Pentland [26] presented an exhaustive search approach across the parameter space, guided by the shading information (but with limited success). Solina and Bajcsy [2,22] addressed the problem of recovering superquadrics from range imaging with the use of least-squares minimization and the inside–outside function, which define the relation between a point in space and the superquadric surface. While alternative methods were also discussed at the time [27,28], they proved to be less efficient (resource-wise), while achieving similar performance [29].

The inside–outside function, due to its success, was later also used by various other researchers [30,31]. An extension of the initial method was proposed a decade later by Leonardis et al. [32], which allowed for the segmentation of complex shapes with the use of multiple superquadrics. Krivic and Solina [33] introduced an object recognition system, which combined the use of interpretation trees with previous image segmentation and superquadric recovery approaches to detect the presence of a predefined object in a scene. Unfortunately, advancements in this field were overall rather slow, due to the computational complexity of the task, which was iterative. In addition, gathering the required training data was difficult due to inefficient data gathering mechanisms.

**Deep Learning Methods.** More recently, the topic of superquadric recovery experienced a resurgence of interest, driven mostly by advancements in deep learning and convolutional neural networks (CNNs). Tulsiani et al. [9] presented a method for reconstructing 3D objects with the use of cuboids as the primitive representations but noted the limits of this representation. Continuing this line of thought, Paschalidou et al. [10] modified the approach to use superquadrics and in turn, achieved substantially better reconstruction results, due to the tremendous range of shapes that superquadrics can represent. However, to train the CNN predictor, this work relied on labeled 3D data, including models of various object categories, such as human bodies or vehicles. This approach was later adapted [11] to recover superquadrics, in a hierarchical and unsupervised manner, given a single RGB image of a 3D model from the predefined categories. Another reconstruction approach was also proposed by Li et al. [15], which achieved segmentation of point cloud data into parts in an unsupervised manner. Despite tremendous advancements, reliance on labeled 3D data limits the applicability of these approaches to arbitrary data.

To address the need for a more generalized solution, Oblak et al. [12] introduced a supervised deep learning approach, inspired by previous work [2], for estimating the superquadric parameters from a single depth image under the constraint that the orientation of the superquadric was fixed. Šircelj et al. [14] built upon this method and achieved segmentation and recovery of multiple superquadrics. A different avenue was taken by Slabanja et al. [34], who focused on reconstruction from point cloud data. Most recently, Oblak et al. [13] extended their previous work by introducing a novel geometry-aware learning objective, which allows the model to also predict parameters of rotated superquadrics. They also proposed two learning strategies, an explicit and an implicit variant. Despite their success, these approaches are limited to either 3D data or depth images. Unfortunately, for arbitrary tasks, both types of data remain rather difficult to obtain, at least when compared to RGB images. To address this gap, we present a solution that is able to recover high-quality superquadric models from only a single RGB image.

### 2.2. Deep Learning and 3D data

Although this work focuses on superquadric recovery from 2D data, some relevant concepts are closely related to recent deep learning techniques designed for 3D data. Below, we discuss the main overlapping topics between the two problem domains, i.e., the choice of data representation and issues related to pose estimation.

**Choice of Data Representation.** To tackle the task at hand, we must first decide how the input data will be represented. In recent work, Wu et al. [35] proposed representing 3D shapes with 3D discretized volumetric grids and presented a deep 3D encoder model, 3D ShapeNet, capable of reconstructing complete 3D shapes given the grid input. In their approach, they also utilized an additional deep model, with which they transformed the initial depth images into 3D volumetric representation. With MarrNet, Wu et al. [36] extended their approach to 2D RGB input images, in which they performed 2.5D sketch estimation, with the use of an encoder–decoder architecture. From the 2.5D data, they then estimated the volumetric grid and the 3D shape of the object with a 3D decoder. While volumetric grids allow for the representation of true 3D data, one of the main shortcomings of such approaches are the 3D encoders, which have a significant impact on system performance and memory consumption.

In comparison, working only with 2D data and 2D encoders drastically reduces system requirements. However, normal 2D images only offer a single perspective of the scene and are thus prone to object self-occlusion and, in turn, loss of information. Recently, Oblak et al. [12,13] proposed reconstructing 3D objects directly from depth images (2.5D data), which encode invaluable 3D information in a 2D structure. Their approach exploits this intrinsic encoding property and only requires 2D encoders to function. To allow for the reconstruction from simple 2D RGB images, we must conquer the challenges presented by the lack of spatial information, such as determining the positions of objects in space.

**Pose Estimation Issues.** The most recent approaches for estimating the position of an object in space use CNN-based models to estimate the pose parameters from a given continuous range of values. For example, Miao et al. [37] estimated all pose-related parameters (position and rotation) with six separate regressors and a mean squared error (MSE) loss. Another example is the work by Zhu et al. [38], who trained their pose regressor alongside a standard volumetric reconstruction encoder–decoder model. Despite the success of these methods, other approaches based on geometry-aware loss functions have been shown to perform better than regular MSE loss-based approaches. A recent approach by Xiang et al. [39], for example, showcased the strength of such approaches, by minimizing the distance between surface points of rotated objects.

To achieve successful pose estimation, it is also important to consider how the rotation of objects is described. While we often associate Euler angles with rotation, they can be rather problematic since they suffer from gimbal lock. In comparison, using quaternions to represent rotation, solves this issue. On the other hand, relying on quaternions typically involves using the unit norm constraint, which slightly complicates the regression task [38]. Oblak et al. [12,13] combined the above-mentioned methods to estimate the size, shape, position, and rotation of superquadrics. Their results showcase the importance of using a geometry-based loss function for training, as well as quaternions over Euler angles for representing the rotation of superquadrics.

## 3. Methodology

In this section, we provide a formal definition of superquadrics and describe the CNN predictor used for superquadric reconstruction. In addition, we describe the loss functions used and training procedure applied to learn the CNN model.

### 3.1. Superquadrics Definition

**Figure 2:**
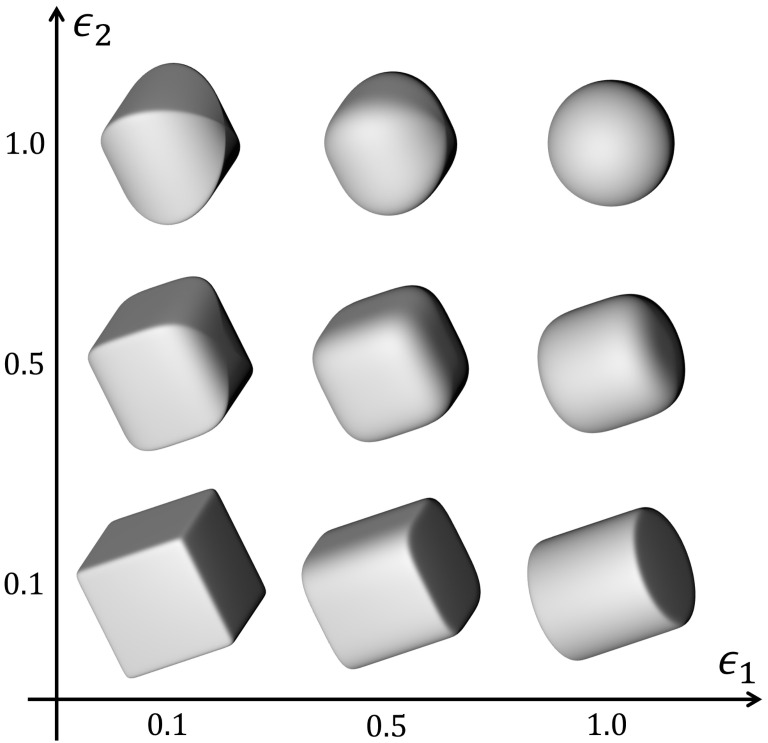
Possible superquadric shapes obtained by changing the shape parameters $\epsilon_{1}$ and $\epsilon_{2}$.

We used superquadrics to represent various volumetric primitives, ranging from spheres to box-like shapes. These are described by the so-called *inside–outside function* F(x,y,z), which is defined for each point in the given space as:

(1) $$F\left(x,y,z\right)={\left({\left(\frac{x}{a_{1}}\right)}^{\frac{2}{\epsilon_{2}}}+{\left(\frac{y}{a_{2}}\right)}^{\frac{2}{\epsilon_{2}}}\right)}^{\frac{\epsilon_{2}}{\epsilon_{1}}}+{\left(\frac{z}{a_{3}}\right)}^{\frac{2}{\epsilon_{1}}},$$

where the input x,y, and *z* parameters determine the position of the superquadric in the space and the three *a* parameters determine its size, with regard to each axis of the coordinate system. For example, $a_{1}$ denotes the size of the superquadric along the *x*-axis and so forth. The two ϵ parameters determine the shape of the superquadric. Their combined effect on the final shape is visualized in Figure 2.

However, such a formulation only defines the superquadric in terms of local or superquadric-centered coordinates. In order to also define the rotation of the object, we must take into account the transformation from world-space coordinates $p_{w}$ to local coordinates $p_{s}$ via $p_{s}=M^{-1}p_{w}$. Note that $p_{w}$ and $p_{s}$ must be represented in the form of homogeneous coordinates. Here, $M^{-1}$ represents the below transformation matrix, where the $r_{i,j}$ values represent elements of a 3×3 rotation matrix and the $t_{i}$ values represent the translation vector, i.e.,

(2) $$M^{-1}=\left[\begin{array}{cccc} r_{11} & r_{21} & r_{31} & -t_{1}r_{11}-t_{2}r_{21}-t_{3}r_{31}\\ r_{12} & r_{22} & r_{32} & -t_{1}r_{12}-t_{2}r_{22}-t_{3}r_{32}\\ r_{13} & r_{23} & r_{33} & -t_{1}r_{13}-t_{2}r_{23}-t_{3}r_{33}\\ 0 & 0 & 0 & 1 \end{array}\right].$$

This transformation allows us to evaluate the inside–outside function in world space coordinates, as $F(M^{-1}p_{w})$. Normally, a camera calibration matrix would also need to be defined to reconstruct objects directly from a 2D image, especially when they are placed at different depths from the camera. However, in this work, we employed a learning-based approach and were thus able to, in a sense, learn or compensate for the camera calibration process, and determine the position of the object in space from various other image cues, such as shading and shadows, with the use of deep learning.

In total, 12 parameters (λ) are required to define a unique superquadric via $F(p_{w};\lambda )$, with $p_{w}$ being the world-centered points and λ the following 12 parameters:

(3) $$\lambda =(a_{1},a_{2},a_{3},\;\epsilon_{1},\epsilon_{2},\;t_{1},t_{2},t_{3},\;q_{1},q_{2},q_{3},q_{4}).$$

To also describe the rotated superquadrics, the four $q_{i}$ parameters were added, representing the coefficients of a unit quaternion. With the Euler–Rodriguez formula [40], these parameters can be used to define a rotation matrix, and at the same time avoid the gimbal lock problem, which Euler angles suffer from.

An incredibly useful attribute of superquadrics and the aforementioned inside–outside function is that we can easily determine if a point lies inside or outside the superquadric. If F(p)<1, then the point is inside and vice versa. In the case that F(p)=1, then the point lies on the surface of the superquadric. This makes the function *F* continuous and differentiable, thus presenting a great foundation for defining a loss function for training neural networks.

### 3.2. Problem Definition and Loss Functions

**Figure 3:**
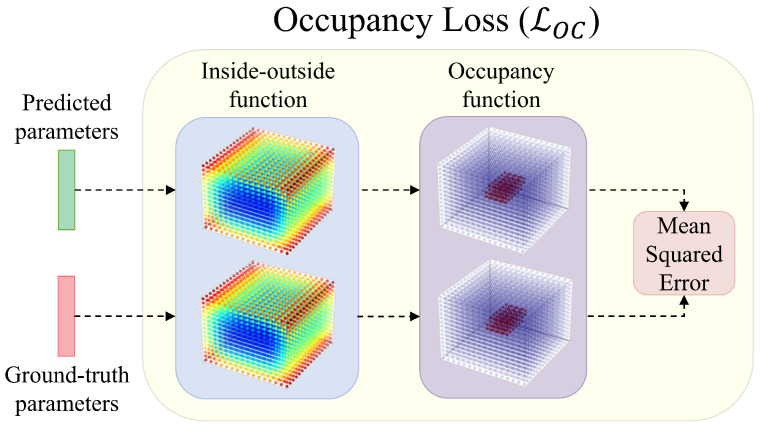
Visualization of the learning objective. To train the CNN predictor for superquadric recovery, we used a geometry-aware loss function based on the 3D occupancy grids constructed from predicted and ground truth parameters.

In this section, we describe one of the main parts of our reconstruction approach from Figure 1, i.e., the CNN predictor. As illustrated in Figure 3, the predictor relies on an occupancy-based loss derived from the inside–outside function. Below, we elaborate on this loss function and provide details on the training procedure used to learn the predictor.

Our goal is to predict, as closely as possible, the λ parameters of a superquadric, based on a single intensity or color image. To obtain these parameter predictions, denoted as $\hat{\lambda }$, we rely on a convolutional neural network (CNN). We also assume that the ground truth parameters λ are given alongside each training image, thus allowing a much easier supervised learning approach for training the model. The superquadric parameters are also continuous real values, so we can think of this task as a sort of regression problem.

To train our neural network, we used a *geometry-aware occupancy loss* function [13]. The occupancy loss was calculated by first evaluating the inside–outside function of the ground truth and the predicted superquadric for every point in space. This resulted in two superquadric hypersurfaces, which could be used as indicators of the training error. To simplify the comparison, we transformed the results of the inside–outside function with an occupancy function *G*, introduced by Paschalidou et al. [11]:

(4) $$G\left(x,y,z\right)=\sigma (s\left(1-F^{\epsilon_{1}}\left(x,y,z\right)\right)),$$

where σ(·) denotes the sigmoid function, which returns values between 0 and 1, and the *s* represents a scaling factor, which controls the sharpness of the spatial border of the superquadric. This parameter is set to 5, based on extensive qualitative experimentation, to ensure that the superquadrics, rendered during training, match their counterparts in the datasets, as closely as possible for the same parameters. Furthermore, the function σ(·) is continuous and, thus, differentiable. It results in values near 0 if a given point is outside the superquadric, 1 if inside, and 0.5 if exactly on the surface. The inside–outside function *F* is also raised to the power of $\epsilon_{1}$ before computing the occupancy function, as suggested by previous work on superquadrics [2,41], in order to combat the overwhelming influence of the shape parameters on the prediction error and to spread the influence more evenly across all parameters. This operation makes the function more suited for convergence, without altering the surface itself.

To speed up the entire procedure, an approximation of the hypersurface was taken by discretizing the space into a set of fixed equally-distanced points *r* on each axis, limited in space by the predefined boundaries $b_{\min}$ and $b_{\max}$ in terms of axis values. Thus, we only had to evaluate the occupancy function for the smaller set of points in the grid $V_{G,\lambda }\left(i,j,k\right)=G\left(x_{i},y_{j},z_{k};\lambda \right)$, with i,j, and *k* ranging from 1 to *r*. The number of points sampled along each axis was directly determined by the resolution parameter *r*, which controlled the balance between the speed of training (lower values) and the smoothness of the loss function (higher values). The final occupancy loss was then defined as the difference between the two occupancy grids in terms of the mean squared error (MSE). This was achieved by summing all of the squared differences of matching points and dividing by the size of the grid. More formally, the occupancy loss is computed as follows:

(5) $$L_{OC}\left(\lambda ,\hat{\lambda }\right)=\frac{1}{\left|V\right|}\sum_{i,j,k}^{r}{\left(V_{G,\lambda }\left(i,j,k\right)-V_{G,\hat{\lambda }}\left(i,j,k\right)\right)}^{2}$$

where λ and $\hat{\lambda }$ correspond to the ground truth and predicted superquadric parameters, respectively.

### 3.3. Neural Network

**Figure 4:**
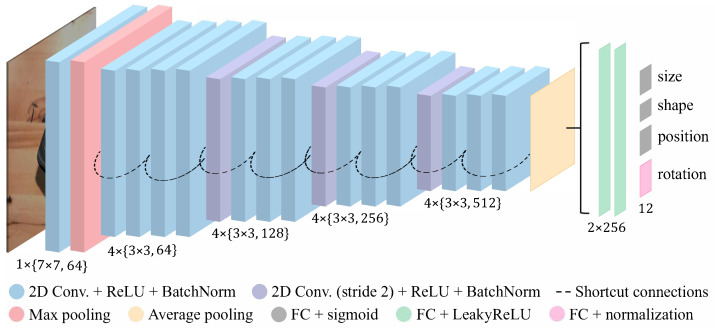
Visualization of the modified ResNet-18 model used for predicting superquadric parameters. The network input is a color image and the outputs are the different superquadric parameter groups. The notation {X×Y,Z} represents a convolutional layer with *Z* filters of size X×Y.

As shown in Figure 1, we used a CNN to predict superquadric parameters from a single color image. While a myriad of different models exists, we used a modified ResNet model, which was shown to perform well on a similar problem of predicting superquadrics from depth images [13]. Using a similar model also allowed us to compare the complexity of the tasks. A shallow ResNet-18 model [42] was chosen as the predictor, due to the simplicity of the input images. The entire network is visualized in detail in Figure 4.

For the starting convolutional layer of our neural network, we relied on a filter size of 7 to obtain a larger receptive field. All following filter sizes were set to 3 and had a stride of 2 every 3 layers, widening the receptive field. We added, to the end of the network, two 256-dimensional fully-connected layers, which combined features observed by the network and captured correlations between spatially distant parts of the generated feature maps. Finally, at the top of the network, we added four output heads, corresponding to the different parameter groups. The sizes, shapes, and positions of the output heads included a fully-connected layer, whose number of outputs depended on the number of parameters in the group, so 3 (size), 2 (shape), and 3 (translation) outputs, respectively. Because the inside–outside function can be unstable with low parameter values, each group ends with a sigmoid activation function, resulting in values closer to 0.5. The fourth and final output head predicts the rotation parameters. It includes a fully-connected layer with four outputs and an $L_{2}$-based normalization activation function. After the predictions were made, the size and position parameters were scaled back to their original range, allowing for the visualization of the predicted superquadrics later on.

### 3.4. Synthetic Data Generation

A key step of our reconstruction approach from Figure 1 is the generation of synthetic data. Without it, training our deep model would be nearly impossible, due to the low availability of annotated superquadric data. Each synthetic data pair includes an image of a single random superquadric along with its parameters. To generate synthetic images that mimic real RGB images captured in various environments, we propose the following flexible generation pipeline.

We began by generating a random superquadric, whose parameters (size, shape, position, rotation) were sampled from uniform distributions, with predefined boundaries, such that they fit in the view frame of the scene. The size parameters corresponded to U(25,75) and the shape parameters to U(0.1,1), to avoid the unstable nature of the inside–outside function, which occurs at low ϵ values. The rotation parameters are represented in the form of quaternions and were sampled from a random uniform rotation, with the use of the subgroup algorithm [43]. The position parameters are based on U(48,208), with an exception of some datasets, for which the *z*-axis position is fixed to an arbitrary value. This was done to enable stable learning of other parameters, since the depth at which the superquadric is placed cannot be easily determined from a non-depth image from the given perspective and coordinate system.

Next, we placed the superquadric in an empty scene of the Pyrender renderer (available at: https://pyrender.readthedocs.io/, accessed on 9 February 2021) and began scene construction. For the needs of our experiments, we generated multiple datasets, whose images mainly differed in the forms of colors and textures. The first and simplest dataset contained intensity images, in which a gray superquadric was displayed in front of a black background. The scene was illuminated by a directional light source and rendered in orthographic projection. This dataset resembled the dataset based on depth images used by Oblak et al. [13], which used the distance from the viewpoint for pixel values.

**Figure 5:**
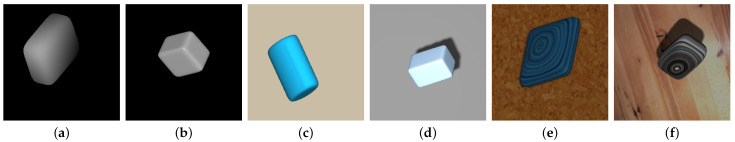
Various images of superquadrics (SQs) generated by the renderer present in Figure 1. (**a**) Depth image; (**b**) intensity image; (**c**) color image; (**d**) color image with shadows; (**e**) textured SQ and background; (**f**) textured SQ on wood with shadows.

More complex datasets are based on RGB images. Some include randomly uniformly colored superquadrics and backgrounds, while others use random textures from a combination of KTH, Kyberge, and UIUC texture datasets (available at: https://github.com/abin24/Textures-Dataset, accessed on 14 September 2021) instead of uniform colors. Due to the difficulties of texturing random objects [44], we applied the textures to our superquadrics in a rather elementary manner, which resulted in symmetrical patterns. For the datasets that sought to emulate realistic images, we resorted to only using manually gathered wooden textures for the background. Additionally, for some datasets, we enabled shadows in the scene, in hopes of providing more spatial information regarding the position of the superquadric. This approach was inspired by similar works [16,17], which showcased the ability to recover shape and size information from shadow-based cues. We also added additional spotlight for some datasets, to better illuminate the scene and cast larger and more realistic shadows, which are hopefully more informative. The new light source was placed randomly in one of the preselected positions around the camera, casting shadows in a random direction. These positions were manually selected along the *x* and *y* axes around the camera, in order to cast meaningful shadows. This approach was chosen because selecting light positions completely at random often resulted in unusable images. We also did not resort to using a single fixed light position, because our main goal was to generate images that would be representative of real uncontrolled environments, for which the light position was not known in advance. Examples of generated images from different datasets can be seen in Figure 5.

## 4. Experiments and Results

This section presents the various experiments conducted to evaluate the proposed superquadric recovery approach. We first briefly describe the experiments and then elaborate on the datasets, performance metrics, and training procedure. Finally, we discuss the results and findings of our experiments.

### 4.1. Experiments

As part of our research, we conducted a series of 11 distinct experiments, in which we learned to estimate superquadric parameters from images of varying complexities. In each of the experiments, we trained a ResNet-18 neural network on a different dataset of images, with the use of the occupancy loss. The first experiments related to intensity images, while later experiments added color and texture to both the superquadrics and the backgrounds. Lastly, we also employed the use of shadows to generate more realistic data. We analyzed and compared the performance of the models trained on different datasets to investigate how different image conditions affect the accuracy of superquadric recovery. In addition, we explored if the models trained on artificial images could be used to recover superquadrics from real images of random objects. Finally, we compared our reconstruction method to the current state-of-the-art method presented by Paschalidou et al. [10].

### 4.2. Datasets

According to the above-described experiments, we generated multiple synthetic image datasets with different attributes for each experiment, ranging from simple intensity images to textured RGB images. All datasets were created as described in detail in Section 3.4. To allow for a fair comparison of results, we used the same randomly sampled superquadric parameters across all datasets, limiting the differences between them. Each dataset included 120,000 images in total, with 100,000 belonging to the training set and the remaining 20,000 being evenly split among the validation and test sets. These values were chosen to ensure even parameter distributions and to provide a sufficient number of images for training the CNN predictor.

### 4.3. Performance Metrics

To evaluate the performance of our neural networks quantitatively, we compared the ground truth parameters to the predicted ones using the mean absolute error (MAE). We report the mean and standard deviation of errors as well as visualize the error distributions across different superquadric parameters.

Unfortunately, visually identical superquadrics can be obtained with differently ordered parameters, due to the ambiguity of the superquadric description [2]. For example, a cuboid with size parameters $a_{1}=1$, $a_{2}=1$, $a_{3}=2$ is visually identical to a cuboid with parameters $a_{1}=1$, $a_{2}=2$, $a_{3}=1$ if it is rotated by 90° around the *x*-axis in the local coordinate system. Similar behavior can also be observed with the shape parameters $\epsilon_{1,2}$. Thus, the prediction order of the shape and the size parameters were rather arbitrary. To allow for a non-ambiguous analysis of results, we first averaged over the size and shape parameter groups. This way, we obtained one value per parameter group $\bar{a}$ and $\bar{\epsilon }$, which we then used when calculating MAE scores.

Rotation parameters display even worse behavior, particularly for spherical superquadrics, where different quaternion values can result in identical superquadrics. Due to this, the MAE measure was completely unreliable for evaluating rotation parameters. To solve this problem we resorted to a geometry-based performance metric. To compare superquadrics in a geometric manner, we used a variant of the intersection over union (IoU) based on the binary occupancy function B(x,y,z), which evaluates the points inside the superquadric as 1 and the points outside as 0. As before, these values were evaluated only on the approximations of the superquadrics to speed up the entire process.

The IoU measure represents the coverage between the generated and the true superquadric. It is computed as the number of points that belong to both superquadrics, divided by the number of points belonging to either one of them. The overlap or coverage between superquadrics is defined on a range of 0 to 1, describing no and full coverage respectively, i.e.,

(6) $$IoU\left(\hat{\lambda },\lambda \right)=\frac{\sum_{i.j,k}^{r}V_{B,\hat{\lambda }}\left(i,j,k\right)\wedge V_{B,\lambda }\left(i,j,k\right)}{\sum_{i.j,k}^{r}V_{B,\hat{\lambda }}\left(i,j,k\right)\vee V_{B,\lambda }\left(i,j,k\right)}.$$

This allowed us to better evaluate the rotation parameters of the superquadrics, because the overlap of superquadrics was robust to orthogonal rotations along the superquadric axes.

### 4.4. Training Procedure

As described in Section 4.2, we split each generated dataset into three parts. For training, we used 100,000 images and 10,000 images for validation. To analyze and test the trained models, we used the remaining 10,000 images. We provided the images as inputs to our modified ResNet-18 model, whose backbone was pretrained on the ImageNet dataset [45]. The entire network was then fine-tuned for the superquadric task. The model outputs were obtained through the sizes, shapes, positions, and rotation output heads, corresponding directly to the superquadric parameters. To train the model, we used the Adam [46] optimizer with an initial learning rate of $10^{-4}$ and the occupancy loss function. This function also depended on a resolution parameter *r*, which we set to r=32, to ensure a balance between the smoothness and the computational complexity of the loss function.

Each epoch consisted of first shuffling the training set, iterating through the entire set, and then evaluating the models at the end. The batch size was set to 32, based on standard methodology [47,48,49]. Our learning rate scheduler also decreases the learning rate by a factor of 10, when the validation loss does not improve for 10 epochs in a row. The entire training procedure is stopped when the validation loss does not improve in the 20 consecutive epochs. For testing, we used the best-performing model on the validation set, in terms of occupancy loss. The superquadric parameters are learned at about the same rate, based on the validation error curves. The model converges in only around 150 epochs on all datasets.

### 4.5. Results

In this section, we present both a quantitative and a qualitative analysis of the results, obtained with identical CNN predictors trained on various datasets. In addition, we compare our findings with previous research conducted on depth images [13]. We split the following sections into three parts. First, we analyze the results obtained on grayscale and color images, also denoted as 2D images. We then discuss the *Fixed z*-axis parameter limitation and ways to solve it. Next, we compare our method with the state-of-the-art method by Paschalidou et al. [10]. Finally, we observe how well our predictors, trained on artificial data, perform on real-life images.

**Figure 6:**
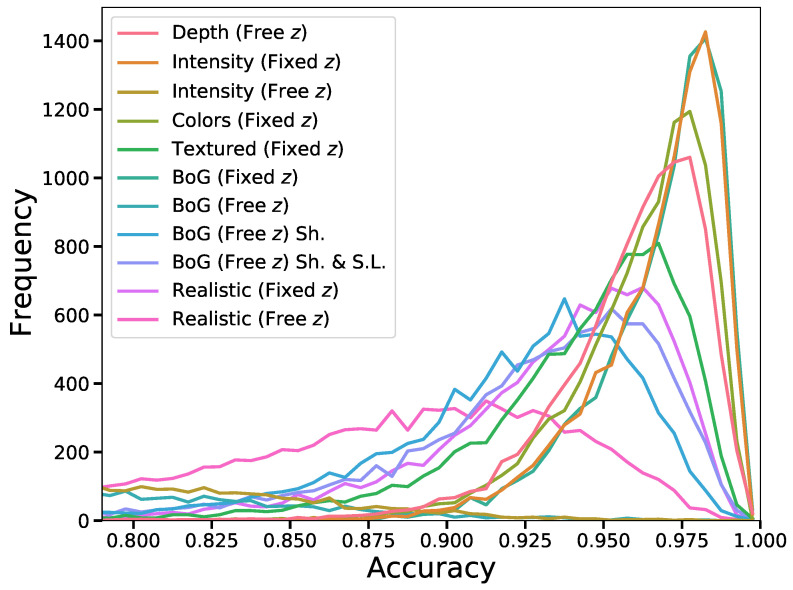
IoU score distributions of CNN predictors trained on different datasets. The distributions are based on predictions of the corresponding test images. Clear performance decay can be observed with increasing complexities of images.

**Figure 7:**
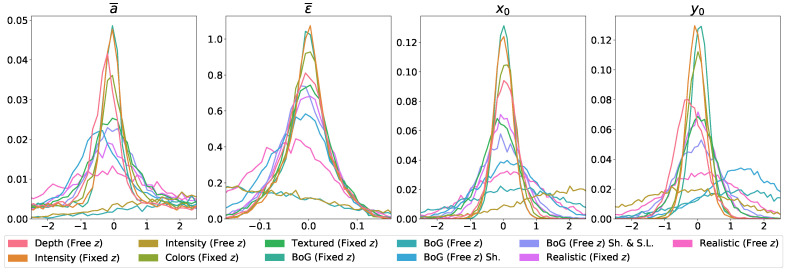
Visualization of mean absolute error (MAE) distributions across the predicted parameters. Each trained model is represented by its own color. Size ($\bar{a}$) and shape ($\bar{\epsilon }$) errors are averaged over all elements of the parameter group, due to the arbitrary ordering discussed before. Errors of the *z* parameter are not reported, because the distributions serve no purpose for most models, due to the *Fixed z* parameter.

We report the obtained mean absolute errors (MAE) and mean IoU score of the CNN predictor on different datasets in Table 1, alongside standard deviation values. Furthermore, we visualize the distributions of IoU accuracies in Figure 6 and the error distributions over the predicted parameters in Figure 7. These figures include the results of all trained models.

#### 4.5.1. Reconstruction from 2D Images

**Results with Intensity Images**. The first set of experiments aims to evaluate the performance of our ResNet-18 model on regular intensity images and compare it to the performance on depth images, to determine whether superquadric recovery is also possible from images that lack spatial information. To ensure a fair comparison with previous work, we made sure that our generated depth images closely resembled those of Oblak et al. [13] and retained the same renderer setup for intensity images. In addition, we generated both datasets using the same superquadric parameters.

By simply comparing the IoU values from the first two rows in Table 1, it is clear that the model trained on intensity images with no restrictions (*Intensity* (*Free z*)) performed considerably worse than the one based on depth images (*Depth*), with the latter scoring 0.387 higher on average. This large discrepancy can be attributed to the enormous errors made when predicting the *z* position parameter. Predicting these values is virtually impossible, at least in the given setting, considering the camera perspective and the superquadric coordinate system. For example, identical images can be obtained with a smaller superquadric placed closer to the camera and a larger superquadric placed further away. This issue, in turn, noticeably affects predictions of other parameters, because the model does not converge properly. Furthermore, this showcases the difference in difficulty between the given task and the one tackled in previous work by Oblak et al. [13].

**Figure 8:**
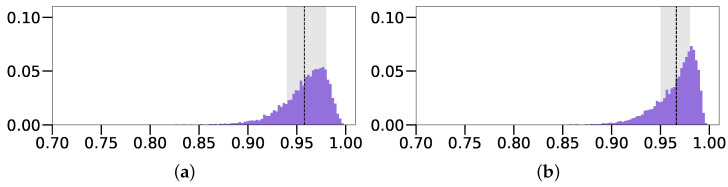
Distributions of IoU values obtained when training the model on regular depth images and on intensity images with the *Fixed z* position requirement. (**a**) Depth; (**b**) Intensity (*Fixed z*).

To combat this issue, without altering the image generation process, we trained our predictor on a dataset of intensity images with a *Fixed z* position parameter (*Intensity* (*Fixed z*)), meaning that it was set to a fixed value across the dataset. With this configuration, our model achieved considerably better performances across the board, in terms of all MAE parameter values and IoU accuracy. It even slightly surpassed the model trained on depth images, which can more clearly be seen in Figure 8, where we see that the distribution of the intensity-based model has a much higher peak and smaller standard deviation range. Its mean IoU value of 0.966 was also slightly higher compared to the 0.958 of the depth-based model of Oblak et al. [13]. In addition, we observe rather low standard deviation values overall, which suggests that the predicted parameters are fairly close to the ground truth for the majority of images. However, this performance comes at the expense of not being able to predict the *z* position parameter, which negatively affects the capabilities of the trained CNN predictor.

By analyzing the error distributions in Figure 7, we observe that both *Depth* and *Intensity* (*Fixed z*) models have rather Gaussian-like error distributions for all parameter groups, centered around an error of 0, exhibiting stable behavior. In comparison, the model based on intensity images with an unlocked *z* parameter (*Intensity* (*Free z*)) exhibits rather unstable behavior with non-Gaussian error distributions that are heavily skewed in either the negative or positive directions.

From this experiment, we conclude that superquadric recovery from intensity images can be just as successful as recovery from depth images [13]. However, this is only true if some form of additional spatial information is provided, such as the *Fixed z* position of superquadrics in space, which determines how far away from the camera the object is. Without this constraint, the position of the superquadric becomes ambiguous and, thus, drastically affects performance.

**Results with Color Images.** Having showcased that superquadric recovery is possible from intensity images, we now focus on the reconstruction from color images. The following experiments aimed to explore how the complexity of color images affects the performance of our CNN predictor.

We begin with a model trained on images with uniformly colored superquadrics and backgrounds, whose superquadrics follow the *Fixed z* parameter constraint as discussed before, denoted as *Colors* (*Fixed z*). The model achieves an IoU score of 0.960±0.026, which is only slightly worse than the ones of the *Intensity* (*Fixed z*) model (0.966±0.022). However, it still performs slightly better than the depth image-based model (0.958±0.026) [13]. The *Colors* (*Fixed z*) model also performs slightly worse than the intensity image-based model in terms of MAE scores of all parameters. However, the differences are rather negligible, especially for the shape and position parameters. Thus we can conclude that additional colors and colored backgrounds do not noticeably impact the performance of the predictor, despite the background and superquadric sometimes matching in color.

Next, we increase the complexity of the images by using randomly textured superquadrics and backgrounds, an example of which can be seen in Figure 5. Analyzing the results, we observe a decrease in performance across almost all metrics. The accuracy of the *Textured* (*Fixed z*) model (0.941±0.038) is considerably worse than that of the previous model (*Colors* (*Fixed z*)), both in terms of mean and standard deviation. The same is true for most shape and position parameters. Interestingly, we observed an improvement in the MAE scores for the size parameters, possibly due to the trade-off between parameters. Overall, the obtained results show that our simple CNN predictor remains highly successful, even on significantly more complex images of superquadrics. Despite the performance being slightly lower than that achieved on intensity and color images, it is still acceptable and comparable to the initial performance on depth images.

**Figure 9:**
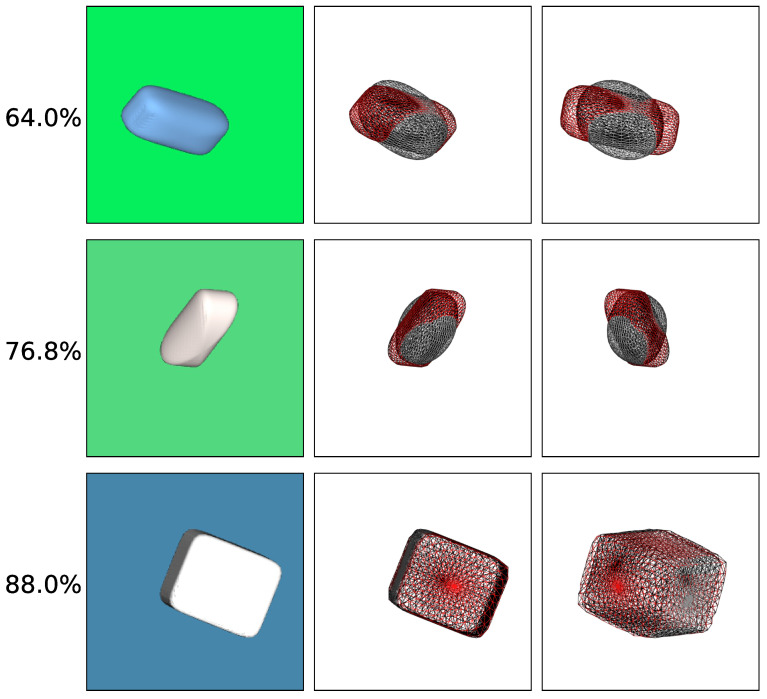

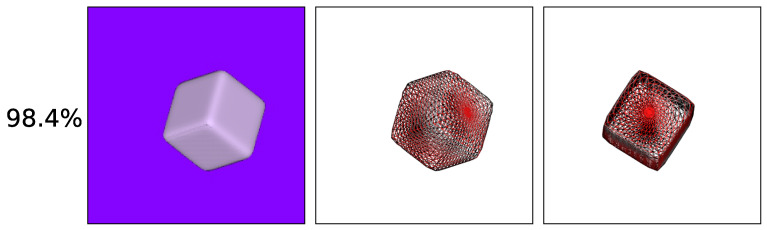
Examples of superquadric reconstructions from across the whole IoU distribution range. The first column depicts input images, while the second and third show the overlap between ground truth (red) and predicted (black) superquadrics in wireframe form, from two different viewpoints.

To better understand the accuracy of our predictions and the errors made, we also present qualitative results achieved with color images. We visualize the superquadric reconstructions of different accuracies in Figure 9. To allow for easier visual comparison, we place both the ground truth superquadric wireframe (red) and the predicted superquadric wireframe (black) in the same scene. We then render the scene from two different points of view, the first being the same as when generating the original images, while the second is slightly moved. As expected, we observe considerable overlap between the wireframes of examples with high accuracy. In comparison, examples with low accuracy overlap quite poorly, which is especially noticeable when depicted from the alternative point of view.

**Figure 10:**
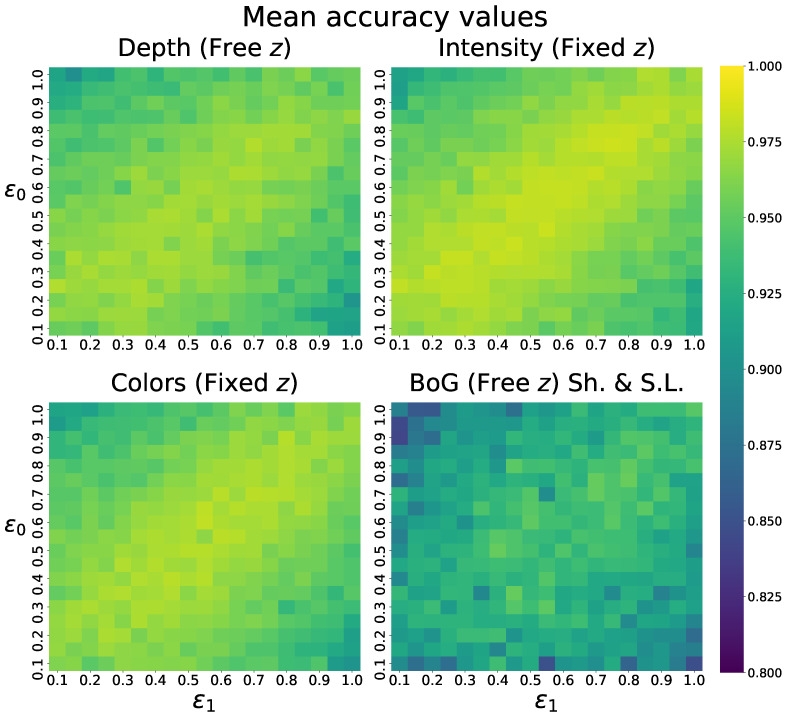
Visualization of mean accuracy values obtained on images of superquadrics with different ground truth shape parameter values.

We also notice an interesting pattern in the qualitative results of this model and others, where the shapes of the superquadrics seem to be related to the obtained accuracy. To analyze this observation in a quantitative manner, we visualize the obtained mean IoU scores of multiple models across the ranges of both ground truth shape parameters $\epsilon_{1}$ and $\epsilon_{2}$ in Figure 10.

From these heatmaps, it is clear that higher mean IoU accuracy is obtained along the diagonal when both shape parameters are fairly similar. Lower IoU accuracy is observed in corners where the two parameters are least similar. This means that our model more accurately predicts superquadrics, which are of symmetrical shapes, such as cubes and spheres, and less accurately predicts non-symmetrical shapes, such as cylinders. We believe that this occurs due to the ambiguity of the superquadric definition, discussed before, since symmetrical shapes allow for reordering of other parameters, without affecting the final superquadric. From this, we can simply conclude that non-symmetrical superquadrics are much more difficult to reconstruct than symmetrical ones, which should be taken into account in future research.

Throughout these experiments, we observed overall extremely positive results. The model based on the randomly colored dataset, with the *z* position constraint, actually still outperforms the model based on depth images [13]. Although the model performs slightly worse on the textured dataset, which is drastically more complex, the performance is still comparable. However, it should be noted that to allow for a more fair comparison, the aforementioned position constraint should first be addressed.

#### 4.5.2. Solving the *Fixed z* Position Requirement

We have shown that our CNN predictor is capable of highly accurate superquadric reconstruction, under the condition that the *z* position in space is fixed. Without this undesirable requirement fulfilled, the accuracy of the reconstructions drops drastically.

To obtain promising reconstructions without additional constraints, we experimented with various possible solutions. In our first approach, we changed the perspective of the camera to an isometric view, prior to the rotation being applied to the superquadric. Unfortunately, this change simply spread the uncertainty across multiple parameters, since the same image could be captured with multiple variations of size and position. For example, the same image could be achieved with a larger object that is positioned further away from the camera.

**Figure 11:**
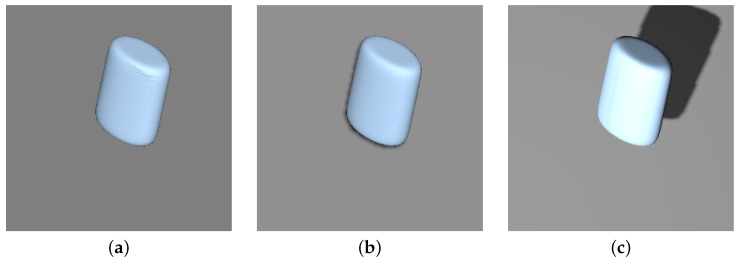
Examples of scene alterations used to counteract the *Fixed z* position requirement. Images depict identical superquadrics with minor scene alterations. The first image is the baseline. In the second, we enabled shadows in the scene. For the third image, we added a spotlight to cast larger shadows. All images contain blue superquadrics on grey backgrounds to allow for better visibility of shadows. (**a**) BoG (*Free z*); (**b**) add shadows; (**c**) add spotlight.

A more successful approach entailed enabling superquadrics to cast shadows on the background object. The added shadows are barely noticeable in the images, as seen in Figure 11, due to the directional light source. In the dataset, the superquadrics are light blue and rendered in front of a gray background (referred to as Blue on Gray, or simply BoG), to allow for better contrast between the object, shadows, and background. This approach was inspired by various research studies [16,17], which showcased the importance of shadows for shape or size estimation. Our idea was that even these minimal shadows and their size differences could help with predicting the *z* position parameter, alleviating the dependence on fixing this parameter.

**Figure 12:**
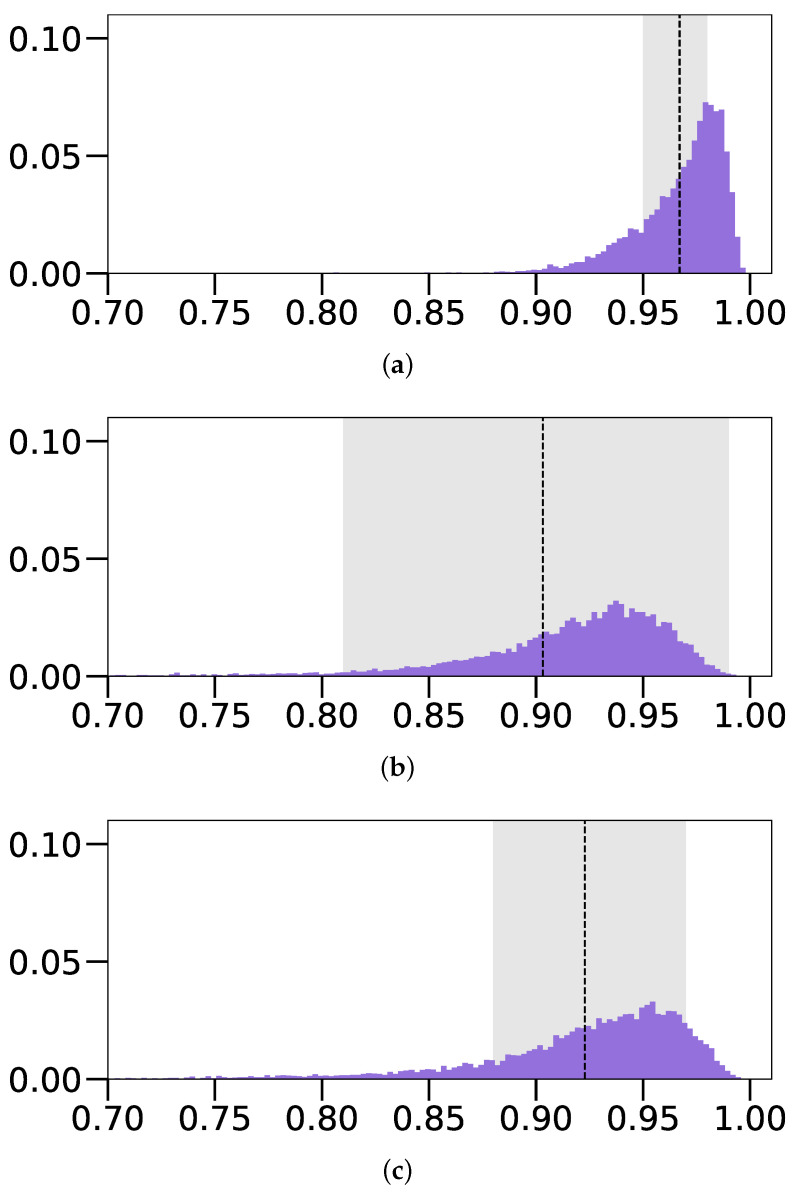
Distributions of IoU values obtained when training the model on variations of the Blue on Gray (BoG) images. (**a**) BoG (*Fixed z*); (**b**) BoG (*Free z*) with shadows; (**c**) BoG (*Free z*) with shadows and spotlight.

Training and testing our CNN predictor on these images, we observed a drastic improvement in IoU scores. The *Blue on Gray (Free z) with Shadows* model achieved a score of 0.903±0.095 in comparison with the results obtained on images without these alterations or the *Fixed z* parameter (0.581±0.180), denoted in Table 1, as *Blue on Gray (Free z)*. We can compare the performance of this approach with the original one, where the *z* parameter is fixed, via the IoU score distributions present in Figure 12. The unrestricted approach with shadows (*Blue on Gray (Free z) with Sh.*) displays a notable performance decrease in both the average IoU score and standard deviation, in comparison with the *Fixed z* variant (0.967±0.023), denoted as *Blue on Gray (Fixed z)*. Observing the MAE scores of both models in Table 1, we notice a drastic increase in position errors, due to the addition of the *z* parameter, while size and shape errors remain fairly similar. Overall, these results reveal that we can bypass the requirement for the *Fixed z* position parameter, at a modest cost of the performance, just by considering barely visible shadows.

In an attempt to further improve these reconstruction results, we added an additional spotlight source (S.L.) to the scene, as described in Section 3.4, thus changing the position, size, and shape of the shadow cast by the superquadric. An example of the described alteration to the superquadric images can be seen in Figure 11. Analyzing the results of the *Blue on Gray (Free z) with Sh. & S.L.* model, we observe an average IoU increase of about 2.0% over the previous model without the spotlight, with the IoU scores being 0.923±0.052. Interestingly, we notice substantially lower MAE values of the *x* and *y* position parameters, as well as a considerable decrease in the standard deviation for all position parameters. Inspecting the MAE distributions in Figure 7, we can see that all distributions of the first BoG-based models without *z* constraints (*Blue on Gray (Free z)*) are heavily skewed, exhibiting rather unstable behavior. The MAE distributions of the second BoG-based model with shadows (*Blue on Gray (Free z) with Sh.*) display drastic improvements; however, some of the distributions are still slightly skewed and not centered around an error of 0. In comparison, the final BoG-based model with the spotlight source (*Blue on Gray (Free z) with Sh. & S.L.*) performs considerably better as all MAE distributions somewhat resemble Gaussian-like distributions. Despite their peaks not being as high as the ones obtained from other *Fixed z* model variants, the distributions are at least centered around the 0 value.

Solving the *z* position constraint also finally allows for a fair comparison between reconstruction from a single RGB image versus a single depth image [13]. Comparing the results of the *Blue on Gray (Free z) with Sh. & S.L.* model and the *Depth* model [13], we only note an accuracy difference of 0.035 in favor of the *Depth* model. This shockingly small difference is impressive from our point of view, especially when considering the clear advantage that the latter approach has, in terms of available spatial information. The reason for the difference is also clearly evident when observing MAE values of the position parameters, where the largest difference is reported for the *z* position parameter, as expected. In comparison, other parameters exhibit considerably smaller differences, especially the shape parameters.

From these results, we can conclude that more prominent shadows, obtained with an additional spotlight source, provide enough spatial information for highly successful reconstruction, without any position constraints. With this change to the artificial images, we are able to train substantially better performing predictors, which are even comparable to the model based on depth images [13].

#### 4.5.3. Comparison with the State-of-the-Art

With the next set of experiments, we compare our superquadric reconstruction method with one of the current state-of-the-art methods introduced by Paschalidou et al. [10], whose work focuses on the reconstruction of complex 3D shapes with multiple superquadrics.

To obtain the required results for the experiments, we relied on the freely available source code (available at: https://github.com/paschalidoud/superquadric_parsing, accessed on 8 December 2021), which accompanies the work by [10]. To allow for a fair comparison of results, we limited the number of superquadrics recovered by the aforementioned method to one. Since this change makes several parts of their method redundant, we also ignored the parsimony loss, responsible for scene sparsity. This model was originally used with voxel form data, such as objects from the ShapeNet dataset, but it also works with other forms of data. Unfortunately, we encountered several problems with convergence when using RGB images, resulting in a rather bad overall performance. Thus, we chose to use the voxel form representation of data, as originally intended by the authors [10]. This decision makes our comparison slightly more difficult since the difference between RGB images (used by our method) and the voxel representation is rather drastic. Most importantly, the latter provides significantly more spatial information, due to our images being limited to a single point of view, which results in the occlusion of some parts of the superquadric. Furthermore, RGB images lack depth information, which was already discussed in previous sections. Due to these differences, we hypothesize that the method by Paschalidou et al. [10] should outperform our method.

To train their model on our synthetic superquadric dataset, we represent each superquadric scene in our datasets with a voxel grid of size (128×128×128), as defined in [10]. The method by Paschalidou et al. [10] also provides users with various learning settings, which we experimented with to obtain the final model. We used a similar learning procedure to the one presented in previous sections and trained the model until convergence.

We report the average and standard deviation values of both methods on two datasets and their subsets in Table 2—these datasets being the intensity dataset with the *Fixed z* parameter and the Blue on Gray (BoG) dataset with the *Free z* parameter, shadows, and a spotlight. We also experimented with subsets of the datasets because the initial experiments of Paschalidou et al. [10] were performed on superquadrics with shape parameters between 0.4 and 1.5, while shape parameters in our dataset ranged from 0.1 to 1.0. To ensure a fair comparison, we trained and tested the two methods first on the entirety of each dataset and then on a subset of the dataset, whose parameters lie between 0.4 and 1.0 as a compromise between the ranges of both papers. This subset included 4417 images of the initial 10,000 image test set.

For the first experiment, we used the dataset based on intensity images of superquadrics with the *Fixed z* position parameter (*Intensity (Fixed z)*). The results of this experiment, reported in Table 2, are rather clear. On the entire dataset, our method achieves considerably better reconstruction performance than the method by Paschalidou et al. [10], with the difference in terms of average IoU scores being 0.168. The latter method also performs worse in terms of standard deviation. Nevertheless, using the entire dataset favors our method, due to the shape parameter range, so we also consider results on the subset of the dataset. The method by Paschalidou et al. [10] displays a larger improvement in IoU scores than our method on the given subset. However, the performance differences between the methods remain quite large (0.159 on average). Interestingly, the method by Paschalidou et al. does not improve in terms of standard deviation, while ours does.

Because we are aware of the effects that such a configuration, with *Fixed z* position parameters, can have on the final reconstruction results, we also trained and tested the two methods on a dataset without this constraint. For this, we used the Blue on Gray dataset with shadows and a spotlight source (*BoG (Free z) with Sh. & S.L.*), in order to provide our model with enough spatial information via shadow-based cues, as discussed in Section 4.5.2. For the method by Paschalidou et al., we again used the voxel representation of superquadric scenes, which provided plenty of spatial information about the position in space. Thus, the method should not have reconstruction issues, despite dealing with the slightly more complex task of properly predicting an additional position parameter.

Despite the clear advantage that the method by Paschalidou et al. has in terms of available information, we observe that our method still performs notably better, both on the entire dataset and its subset. Nevertheless, the method by Paschalidou et al. was not noticeably affected by the lack of the *z* position parameter restriction. On average, the IoU score was reduced by 0.024 and 0.026 for the entire dataset and its subset, respectively. In comparison, the performance of our method was impacted heavily by this change, despite the addition of shadows. This can be seen in the decrease of the average IoU scores by 0.043 and 0.040, respectively, alongside major increases in standard deviation values. Overall, we observe that the difference in model performance is slightly smaller on the dataset without the *z* position parameter restriction. However, the difference is still clearly evident.

Nevertheless, it should also be taken into consideration that the method by Paschalidou et al. [10] was not explicitly designed for the reconstruction of simple objects with a single superquadric, but rather for reconstruction of complex objects with multiple superquadrics. Despite this, the above analysis still showcases the power of our reconstruction method among current state-of-the-art approaches on the task of reconstructing simple objects. In turn, it also shows the potential of using our method for future practical approaches, such as robot grasping.

#### 4.5.4. Performance of Different Backbone Architectures

To evaluate the choice of backbone architectures, used for our CNN predictor, we compare the performance of the predictor using two different backbone networks, namely the ResNet-18 [42] and the Inception-V3 network [50], in terms of reconstruction accuracy. Similar to previous experiments, we evaluated the performance of both variants on two datasets, *Intensity (Fixed z)* and *Blue on Gray (Free z) with Sh. & S.L.*, whose images differ drastically in complexity. To allow for a fair comparison, we trained both variants of the CNN predictor under identical training conditions, following the description in Section 4.4, and trained and tested them on the same datasets. We report the average IoU score and standard deviation values achieved by the two different backbone architectures on the two datasets in Table 3.

For the first experiment on intensity images with a *Fixed z* position parameter (*Intensity* (*Fixed z*)), the model with the ResNet-18 backbone architecture clearly outperforms the Inception-V3 variant in terms of reconstruction accuracy, with the difference of average IoU scores being 0.360. Furthermore, the ResNet-18 variant also achieves a drastically lower standard deviation (only 0.022), meaning that this model performs more consistently across a wide range of superquadrics. In comparison, the standard deviation of the Inception-V3 variant is incredibly high (0.146), showcasing that the predictions across the entire test dataset are clearly not as stable, despite being rather successful on average.

In more complex images, namely the *Blue on Gray (Free z) with Sh. & S.L.* dataset, we observe that the ResNet-18 variant still outperforms the Inception-V3-based one. However, it should be noted that the performance of the ResNet-18 variant decreases considerably, by 0.043 on average, while the performance difference of the Inception-V3 variant is not as drastic, only 0.010 on average. More interestingly, while the standard deviation of the ResNet-18 variant increases with more complex images, as expected, it decreases for the Inception-V3 variant. However, the ResNet-18 variant still outperforms the latter.

Despite having significantly more trainable parameters (24 million), the Inception-V3 network still performs considerably worse, overall, than the ResNet-18 variant, with only 11 million trainable parameters. We speculate that this might be due to the over-complication of the mapping between the input and output of the Inception-V3 variant. This is solved by skip connections in the ResNet-18 network, which allow for simple mappings and also address the vanishing gradient problem during training. This in turn also explains why the difference in performance between the two models is noticeably lower on the more complex dataset, as the strong shadows in the images necessitate a more complex mapping and a larger network. Furthermore, since the network is wider and uses multiple kernels of various sizes within the same layer, it should be more suitable for gathering both global and local features from more complex images. Nevertheless, considering all the aforementioned observations, we conclude that for the task at hand the ResNet-18 backbone architecture is the most appropriate, due to its high reconstruction accuracy and efficient training. However, the inclusion of the wider Inception-V3 backbone could prove useful in future research, especially with the transition to larger and more complex images.

#### 4.5.5. Performance on Real Images

**Figure 13:**
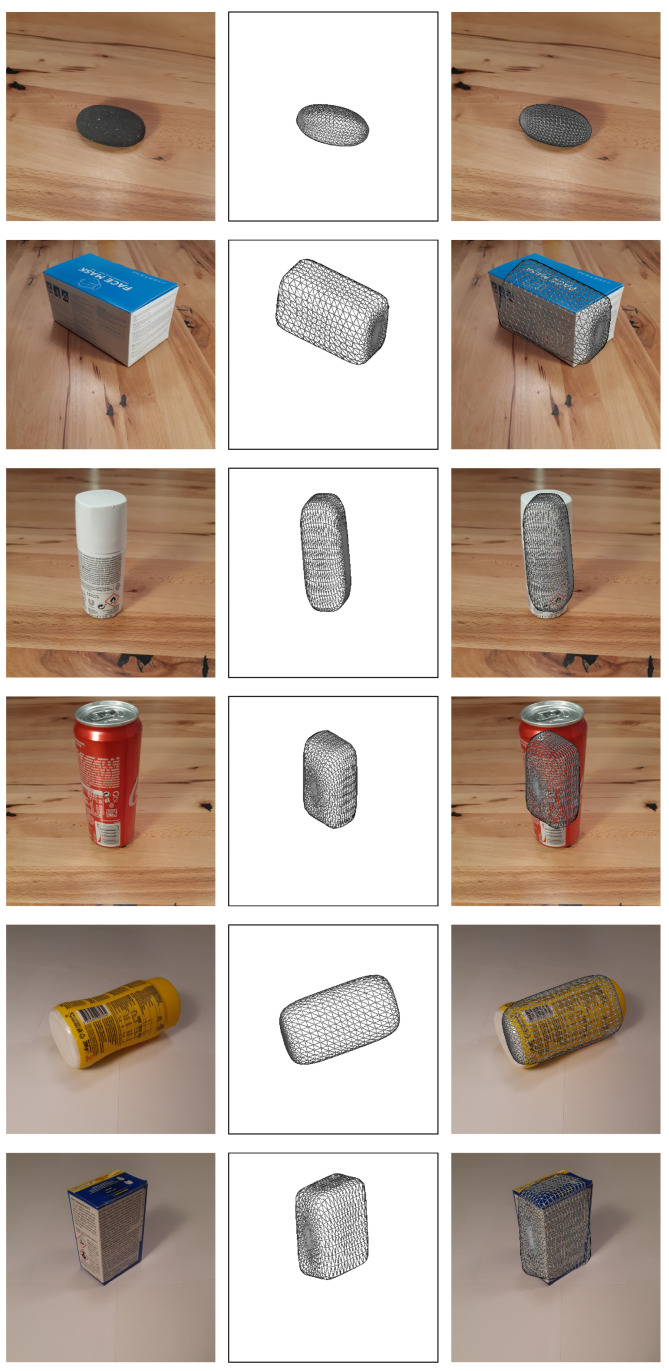
Qualitative results on real images obtained with our final CNN predictor trained on artificial data (*Textured on Wood (Fixed z) with Sh. & S.L.*). The first column depicts the input images, while the second column shows the predicted superquadric in a wireframe form. The last column combines the two images for easier evaluation.

Finally, we analyzed the performance of our reconstruction method on real-world data. To do this, we trained the CNN predictor on synthetic images and tested its performance on real images. First, we captured images of various objects with a phone camera, on a wooden and a white background, and then resized the images to fit the input of our CNN predictors. Examples of the real images used are present in the first column of Figure 13.

To recover superquadrics from these real images, we first tested all trained models discussed above. However, we observed little to no success, as was expected, due to immense differences between the training and testing data. For example, real images included significantly more detailed textures of objects and backgrounds, in addition to the slight difference in projection style. Furthermore, most objects cannot be perfectly described by a single superquadric, due to their more complex shapes. Thus, we decided to construct a new training dataset that would mimic the captured images. To obtain more realistic images, we replaced the vast variety of textured backgrounds with real wooden textures, captured in the same location as the real images. To ensure some variety in training samples, we used 5 different images of the wooden texture. For each generated image, we randomly selected one texture image for the background and then randomly rotated it. In addition, we allowed the superquadric to cast shadows on the background and used the spotlight source. For this task, we constructed two datasets, with one following the *Fixed z* position constraint and another without this constraint, to explore which configuration performed better with real images.

Having trained the two new models, denoted in Table 1 as *Textured on Wood (Fixed z) with Sh. & S.L.* and *Textured on Wood (Free z) with Sh. & S.L.*, we first tested them in a similar fashion as before on the test datasets. We observed fairly high IoU scores for the above-mentioned *Fixed z* variant (0.932±0.042), which were slightly worse, both in terms of average and standard deviation, than the scores of the previous *Textured* (*Fixed z*) model (0.941±0.038). Interestingly, as can be seen in Table 1, the new model (*Textured on Wood (Fixed z) with Sh. & S.L.*) achieved worse MAE scores for the size and shape parameters, but better results for the position parameters, which most likely contributed to the differences in IoU scores. This reveals that the addition of shadows in the scene alongside more realistic backgrounds can negatively impact the performance of our simple CNN predictor, despite being necessary to approach realistic images. In comparison, we observed a considerable drop in performance with the *Free z* variant (last row in Table 1), which achieved an IoU score of 0.858±0.091. However, it should be noted that it did predict all parameters, showcasing that relying on the *Fixed z* requirement is not necessary. Unfortunately, this model (*Textured on Wood (Free z) with Sh. & S.L.*) did not perform as well as the model in the previous section (*Blue on Gray (Free z) with Sh. & S.L.*), despite the main difference only being the textures. We observed that these textures drastically affected the shading and shadows of certain superquadrics. This might be the reason for the performance difference since such information is crucial for the CNN predictor when faced with the *Free z* configuration. We believe this is the reason why the majority of *Fixed* and *Free z* models trained on uniformly colored images, discussed in previous sections, performed considerably better than their *Textured on Wood* counterparts.

Finally, we tested both *Textured on Wood* models on the gathered real images and noted that the *Free z* configuration performed very poorly. In comparison, the model trained on the *Fixed z* image configuration performed fairly well, at least based on qualitative results, displayed in Figure 13. Here, the first column depicts the original image and the second shows the wireframe of the predicted superquadric. By inspecting the last column, which overlaps the wireframe over the input image, we observe that in quite a few of the examples the wireframe fits the captured object quite nicely. Interestingly, being trained on synthetic images with wooden backgrounds, the model (*Textured on Wood (Fixed z) with Sh. & S.L.*) also performs incredibly well on real images with white backgrounds. In our experiments, we observe that, despite different backgrounds, the model subjectively outperforms all others, even those trained on images with uniformly colored backgrounds. This might be caused by the evident shading differences between realistic white backgrounds and artificially colored ones. Despite numerous successful reconstructions, there still exist quite a few suboptimal reconstruction examples for both background configurations. These examples showcase that the model remains rather unpredictable in real images. This could be due to slightly more complex shapes of real objects that superquadrics cannot recreate. Another possible reason could be the slight change in image projection since the model was trained on images rendered in orthographic projection, while real images were captured in perspective projection.

Results of this experiment show that our approach is able to generalize well from artificial training data to real-world data, and is able to successfully reconstruct various simple objects, despite clear differences between the training and testing data. Based on our testing, we believe that the model could also generalize to different real-world scenes if the training images include even more background variety. Overall, these real-world experiments showcase the potential of our approach for future practical applications, for example, for robots grasping unknown objects based on a single RGB image.

## 5. Conclusions

In this paper, we addressed the problem of recovering superquadrics from intensity and color images of varying complexities. Our proposed method extends the method presented in previous research on superquadric recovery from depth images [13]. In our work, we showcase that recovery of superquadrics is also possible from 2D images and that it can be just as accurate as recovery from depth or 2.5D images, despite the lack of spatial information. To achieve this, we propose modifying the training images and ground truth parameters in one of two ways, either by fixing one of the position parameters or by introducing shadows into the scenes. With both approaches, our method achieves considerably better reconstruction results on synthetic images than the current state-of-the-art methods [10]. Additionally, we show that our model can generalize well from synthetic data to real-world images and is able to reconstruct simple unknown objects with a single superquadric. However, performances on real images can be rather unpredictable and require custom synthetic datasets that mimic the given environment.

Our findings showcase the potential of using a deep learning approach based on superquadrics for the 3D reconstruction of unknown objects from a single 2D image, without the need for camera calibration. There exist a myriad of possible future directions. As a next step, we test our approach on real-world tasks, such as a robot grasping random objects, where the *Fixed z* position assumption might be a good approximation of real-world conditions. We believe it would be possible to obtain successful reconstructions even in new environments with minimal human interactions, by simply fine-tuning the model on newly generated synthetic images, whose background matches the new environment. Another avenue of future research includes superquadric recovery from more than one image, for example, from a multi-view camera setup, which could provide necessary spatial information to improve the overall accuracy of our method. Performances on real data could also be improved by texturing synthetic superquadrics more realistically. This work could also be expanded to support the recovery and segmentation of multiple superquadrics to describe more complex objects.

## Figures and Tables

**Table 1:** The table reports the mean and standard deviation values of the mean absolute error(s) (MAE) and IoU accuracies of the predictions on the test set. Size ($\bar{a}$) and shape ($\bar{\epsilon }$) errors are averaged over all elements of the parameter group, due to the arbitrary ordering discussed before. Some *z* errors are crossed out for clarity since some datasets included *Fixed z* parameter values. The abbreviations Sh. and S.L. denote the ability to cast shadows and the addition of a spotlight source, respectively.

Experiment Dataset	Size [0–255]	Shape [0–1]	Position [0–255]			IoU [%]
	$\bar{a}$	$\bar{\epsilon }$	x	y	z	
Depth (Oblak et al. [13])	3.799±4.060	0.040±0.034	0.329±0.305	0.427±0.334	1.359±1.205	0.958±0.026
Intensity (*Fixed z*)	3.773±4.075	0.035±0.031	0.238±0.199	0.239±0.207	/	0.966±0.022
Intensity (*Free z*)	7.670±5.316	0.229±0.154	4.898±9.109	5.164±11.330	33.027±22.427	0.591±0.203
Colors (*Fixed z*)	3.814±4.031	0.037±0.032	0.283±0.232	0.277±0.239	/	0.960±0.026
Textured (*Fixed z*)	3.526±3.850	0.043±0.037	0.484±0.431	0.463±0.420	/	0.941±0.038
Blue on Gray (*Fixed z*)	3.823±4.072	0.036±0.032	0.225±0.186	0.240±0.206	/	0.967±0.023
Blue on Gray (*Free z*)	8.071±4.876	0.236±0.157	3.871±9.460	5.646±11.451	33.199±22.569	0.581±0.180
Blue on Gray (*Free z*) with Sh.	3.836±3.852	0.059±0.053	1.088±3.364	2.312±7.799	6.515±12.180	0.903±0.095
Blue on Gray (*Free z*) with Sh. & S.L.	3.915±4.044	0.046±0.039	0.657±1.166	0.714±1.303	6.063±5.433	0.923±0.052
Textured on Wood (*Fixed z*) with Sh. & S.L.	3.883±3.839	0.046±0.039	0.451±0.390	0.455±0.391	/	0.932±0.042
*Textured on Wood (Free z) with Sh. & S.L.*	4.101±3.860	0.081±0.066	1.357±3.280	1.424±3.686	12.003±11.768	0.858±0.091

**Table 2:** Comparison with the state-of-the-art method of Paschalidou et al. [10] in terms of reconstruction accuracy. The table reports the mean and standard deviation values achieved on two different datasets and their subsets (labeled Sub.). The abbreviations Sh. and S.L. denote the addition of shadows (Sh.) and a spotlight (S.L.) source, respectively.

Experiment Dataset	Method	IoU
Intensity (*Fixed z*)	Ours	0.966 ± 0.022
	Paschalidou et al. [10]	0.798±0.067
Sub. Intensity (*Fixed z*)	Ours	0.972 ± 0.018
	Paschalidou et al. [10]	0.813±0.069
*BoG (Free z) with Sh. & S.L.*	Ours	0.923 ± 0.052
	Paschalidou et al. [10]	0.774±0.068
Sub. *BoG (Free z) with Sh. & S.L.*	Ours	0.932 ± 0.044
	Paschalidou et al. [10]	0.787±0.067

**Table 3:** Comparison of two backbone network architectures for our CNN predictor, in terms of reconstruction accuracy. The table reports the mean and standard deviation values achieved by both predictors on two different datasets. The abbreviations Sh. and S.L. denote the addition of shadows (Sh.) and a spotlight (S.L.) source, respectively.

Experiment Dataset	Architecture	IoU
Intensity (*Fixed z*)	ResNet-18	0.966 ± 0.022
	Inception-V3	0.930±0.146
*BoG (Free z) with Sh. & S.L.*	ResNet-18	0.923 ± 0.052
	Inception-V3	0.913 ± 0.108

## Data Availability

Not applicable.
